# Symptom‐based case definitions for COVID‐19: Time and geographical variations for detection at hospital admission among 260,000 patients

**DOI:** 10.1111/irv.13039

**Published:** 2022-09-05

**Authors:** Joaquin Baruch, Amanda Rojek, Christiana Kartsonaki, Bharath K. T. Vijayaraghavan, Bronner P. Gonçalves, Mark G. Pritchard, Laura Merson, Jake Dunning, Matthew Hall, Louise Sigfrid, Barbara W. Citarella, Srinivas Murthy, Trokon O. Yeabah, Piero Olliaro, ISARIC Clinical Characterisation Group

**Affiliations:** ^1^ ISARIC, Pandemic Sciences Institute University of Oxford Oxford UK; ^2^ Centre for Integrated Critical Care University of Melbourne Melbourne Australia; ^3^ MRC Population Health Unit, Clinical Trials Service Unit and Epidemiological Studies Unit University of Oxford Oxford UK; ^4^ Apollo Hospitals Chennai India; ^5^ The George Institute for Global Health New Delhi India; ^6^ Infectious Diseases Data Observatory, Centre for Tropical Medicine and Global Health University of Oxford Oxford UK; ^7^ Big Data Institute University of Oxford Oxford UK; ^8^ Faculty of Medicine University of British Columbia Vancouver Canada; ^9^ National Public Health Institute of Liberia Paynesville Liberia

**Keywords:** case definitions, COVID‐19, hospitalisation

## Abstract

**Introduction:**

Case definitions are used to guide clinical practice, surveillance and research protocols. However, how they identify COVID‐19‐hospitalised patients is not fully understood. We analysed the proportion of hospitalised patients with laboratory‐confirmed COVID‐19, in the ISARIC prospective cohort study database, meeting widely used case definitions.

**Methods:**

Patients were assessed using the Centers for Disease Control (CDC), European Centre for Disease Prevention and Control (ECDC), World Health Organization (WHO) and UK Health Security Agency (UKHSA) case definitions by age, region and time. Case fatality ratios (CFRs) and symptoms of those who did and who did not meet the case definitions were evaluated. Patients with incomplete data and non‐laboratory‐confirmed test result were excluded.

**Results:**

A total of 263,218 of the patients (42%) in the ISARIC database were included. Most patients (90.4%) were from Europe and Central Asia. The proportions of patients meeting the case definitions were 56.8% (WHO), 74.4% (UKHSA), 81.6% (ECDC) and 82.3% (CDC). For each case definition, patients at the extremes of age distribution met the criteria less frequently than those aged 30 to 70 years; geographical and time variations were also observed. Estimated CFRs were similar for the patients who met the case definitions. However, when more patients did not meet the case definition, the CFR increased.

**Conclusions:**

The performance of case definitions might be different in different regions and may change over time. Similarly concerning is the fact that older patients often did not meet case definitions, risking delayed medical care. While epidemiologists must balance their analytics with field applicability, ongoing revision of case definitions is necessary to improve patient care through early diagnosis and limit potential nosocomial spread.

## 1 INTRODUCTION

Leading public health bodies publish case definitions during disease outbreaks to standardise data collection and inform policy and clinical practice. For, coronavirus disease 2019 (COVID‐19), the World Health Organization (WHO), the European Centre for Disease Prevention and Control (ECDC), UK Health Security Agency (UKHSA, formerly Public Health England, PHE) and the Centers for Disease Control (CDC), United States, have all developed independent case definitions.^1^, ^2^, ^3^, ^4^

At the point of hospital admission, case definitions are used in various ways. They are used to triage suspected patients and isolate them from shared waiting rooms while waiting for laboratory test results to prevent contamination.^5^ Even in well‐resourced healthcare settings, testing turnaround times can be several hours—during which patients are at increased risk of nosocomial transmission if they are not correctly triaged. Case definitions are also used to determine the threshold for laboratory testing in settings where universal testing at hospital admission is not routine or possible. Early during an epidemic, when diagnostic tests may not be available, or in low‐resource areas with limited laboratory diagnostic capacity, using the most appropriate case definition for the local context can aid in making a clinical diagnosis and determine treatment.

Resource allocation may also be impacted by case definitions when they are used to count the number of suspected cases that a healthcare service receives. Similarly, clinical case definitions have been developed for post‐COVID‐19 condition (‘long COVID’),^6^ given that no laboratory diagnostic test exists. If case definitions perform poorly, this can lead to an unacceptable rate of misdiagnosis that risks patient and staff safety.

Clinical case definitions for infectious diseases usually have two components: clinical (e.g., symptoms at the time of presentation) and epidemiological (e.g., contact with a confirmed case). Epidemiological criteria are viewed as dynamic, changing throughout epidemics; for example, in the early months of the COVID‐19 pandemic, many institutions made frequent updates to their travel history criteria as COVID‐19 spread. At the same time, the extent to which clinical presentation is a dynamic phenomenon is complex. The clinical presentation of disease may evolve for reasons including increased knowledge of the clinical characteristics, a relative change in the attack rate in different population groups over time, the influence of new variants and modifying effects of vaccines. Other contributing factors are geographical variation in presenting symptoms due to differences in populations' age pyramids, healthcare access, the prevalence of comorbidities and cultural variations in expressions of symptoms. Concurrent case definitions with different criteria will inherently complexify reporting, benchmarking and research data harmonisation.

Therefore, our objective was to evaluate temporal and geographical patterns in the proportion of hospitalised COVID‐19 patients meeting frequently used COVID‐19 case definitions in the ISARIC (International Severe Acute Respiratory and Emerging Infections Consortium)^7^ database.

## 2 METHODS

### 2.1 Population, setting and study design

The study population consisted of hospitalised patients with laboratory‐confirmed SARS‐CoV‐2 infection reported to the ISARIC database by partner institutions between January 2020 and December 2021. The study design of this prospective, multicountry, cohort study has been described elsewhere.^7^ To be eligible, patients had to have complete information on age, date of admission, country and symptoms on admission. Patients with incomplete outcomes, for example, lost to follow‐up or ongoing care, were removed from analyses of case fatality ratio (CFR) by case definition (see below).

### 2.2 Variables and outcomes

We calculated the percentages of patients meeting case definitions developed by international (WHO, ECDC) and national (UKHSA, US‐CDC) health agencies and how these varied with time (by quarter and year), age (in 10‐year groups) and region (according to the World Bank classification [https://data.worldbank.org/country]), restricted to Europe and Central Asia, South Asia, and East Asia and Pacific. To avoid bias in reporting of patients, regions with less than 8000 patients were excluded. All case definitions are listed in Appendix S1. Because case definitions evolved over time, patients were assessed using the case definition in place when they were admitted (Appendix S1).

Briefly, current case definitions were as follows:

CDC: Acute onset or worsening of at least *two* of the following symptoms or signs: fever (measured or subjective), chills, rigours, myalgia, headache, sore throat, nausea or vomiting, diarrhoea, fatigue, congestion or runny nose. *OR* Acute onset or worsening of any *one* of the following symptoms or signs: cough, shortness of breath, difficulty breathing, olfactory disorder, taste disorder, confusion or change in mental status, persistent pain or pressure in the chest, pale, grey or blue‐coloured skin, lips or nail beds, depending on skin tone, inability to wake or stay awake.
ECDC: At least one of the following symptoms: cough, fever, shortness of breath, sudden onset of anosmia, ageusia or dysgeusia.
WHO: Acute onset of fever *AND* cough; *OR* Acute onset of *any three or more* of the following: Fever, cough, general weakness/fatigue, headache, myalgia, sore throat, coryza, dyspnoea, anorexia/nausea/vomiting, diarrhoea and altered mental status.
UKHSA: New continuous cough, *or*, temperature ≥ 37.8°C, *or*, loss of, or change in, anosmia or taste ageusia.

### 2.3 Statistical analysis

Model‐adjusted CFR for patients meeting or not meeting each case definition were estimated using logistic regression with marginal standardisation methods.^8^ For each model (WHO, ECDC, UKHSA and US‐CDC), dead or discharged alive was modelled as an outcome. Case definition result (met/not met), time (by quarter and year), age (in 10‐year groups), sex and region (according to the World Bank classification) were used as fixed effects. Lastly, symptoms and comorbidities were explored descriptively to evaluate the clinical presentation among those who did not meet the case definitions.

## 3 RESULTS

### 3.1 Inclusion and demographics

**FIGURE 1:**
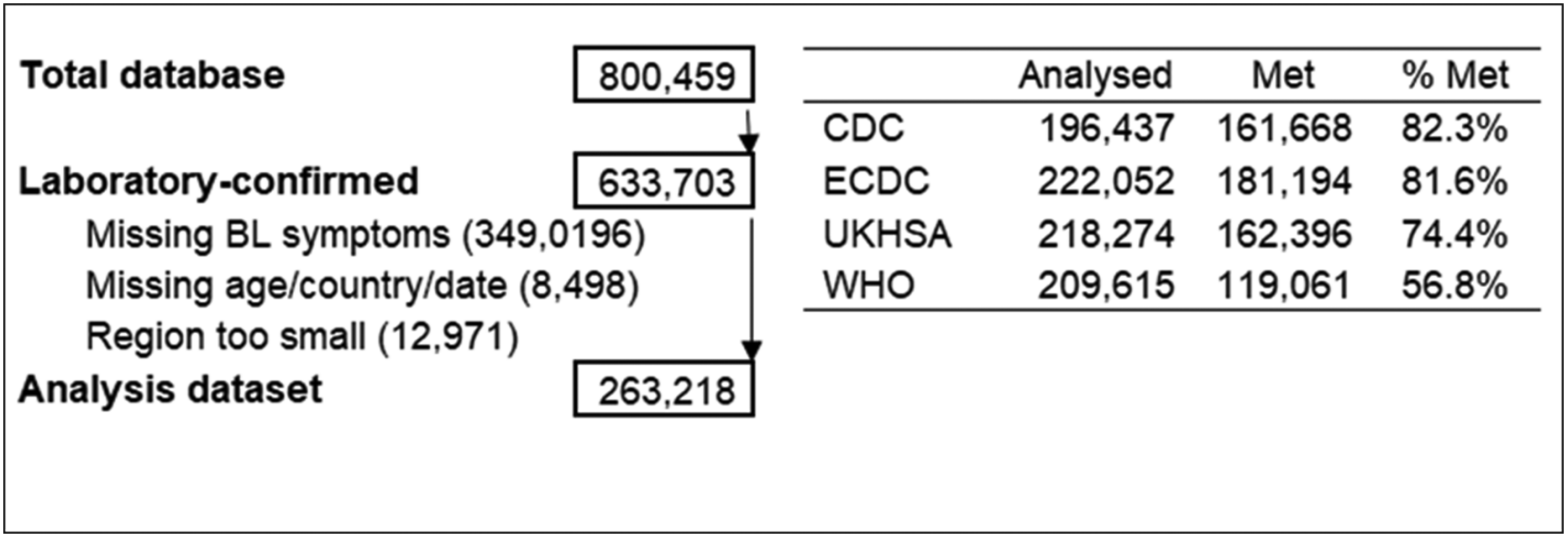
Flowchart of inclusion and exclusion of patients to evaluate commonly used COVID‐19 case definitions among COVID‐19‐hospitalised patients in the ISARIC database (800,459 patients). BL, baseline symptoms not recorded by site; CDC, Centers for Disease Control, United States; ECDC, European Centre for Disease Prevention and Control; UKHSA, UK Health Security Agency (formerly Public Health England, PHE); WHO, World Health Organization

The database analysed includes 263,218 patients, representing 42% of all patients with confirmed Sars‐CoV‐2 infection in the ISARIC database (Figure 1, including reasons for exclusion). Patients included in the analyses and the percentage of those meeting each case definition are shown in Figure 1. The median age was 67 (interquartile range = 29); 43.7% of patients were female. Of these, 238,102 (90.4%) were from Europe and Central Asia, 17,043 (6.5%) from South Asia and 8073 (3.1%) from East Asia and the Pacific. The highest proportion of patients presented during the last quarter of 2020 (23.8%), followed by the second quarter of 2020 (21.7%). The lowest number of patients had data recorded during the second quarter of 2021 (3.6%).

### 3.2 Case definitions

**FIGURE 2:**
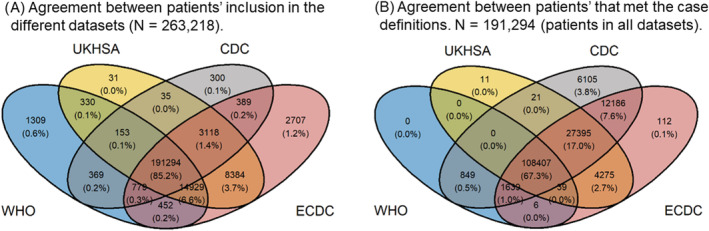
Agreement between datasets (panel A) and agreement between patients meeting the case definitions (panel B) in an analysis of the commonly used COVID‐19 case definitions among COVID‐19‐hospitalised patients in the ISARIC database

**FIGURE 3:**
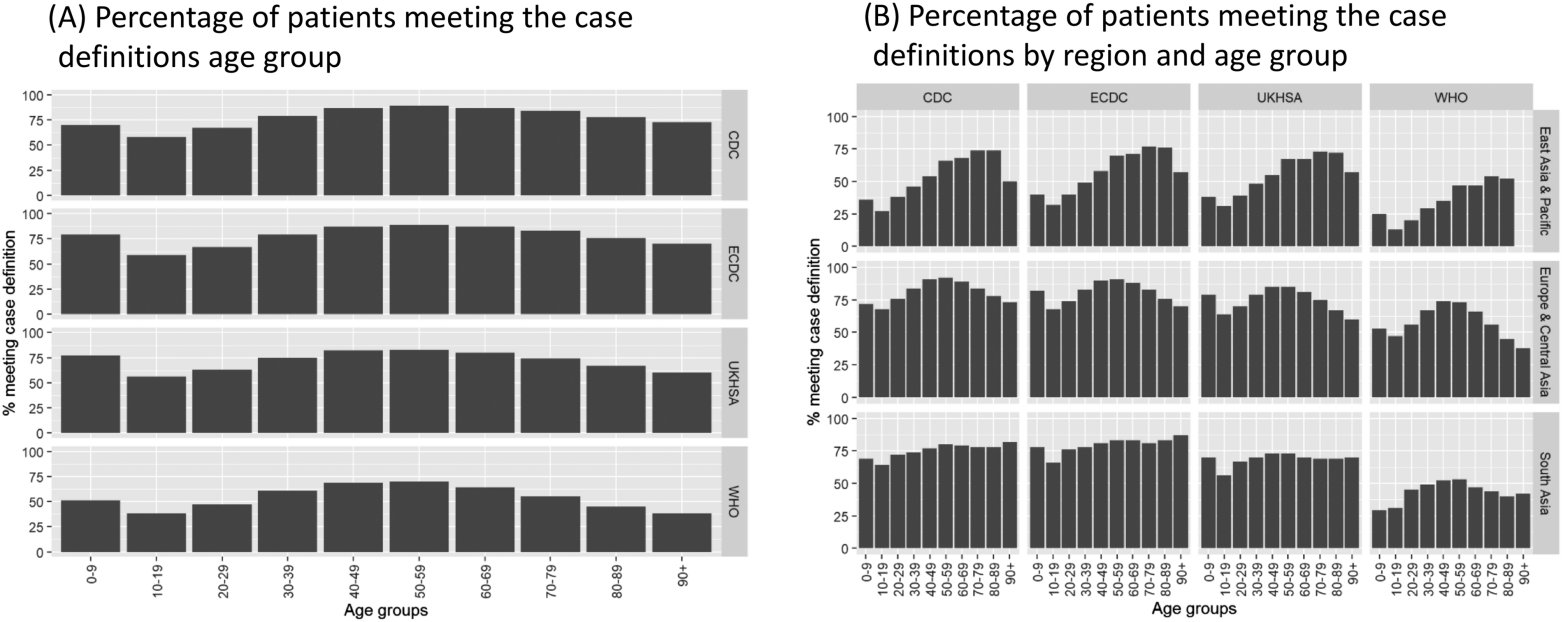
Percentage of patients in the ISARIC database meeting the United States CDC (N = 196,437), ECDC (N = 222,052), UKHSA (N = 218,274) and WHO (N = 209,615) case definitions by age and geographical region. Panel (A), patients by age. Panel (B), patients by age and geographical region (using the World Bank classification). CDC, Centers for Disease Control, United States; ECDC, European Centre for Disease Prevention and Control; UKHSA, UK Health Security Agency (formerly Public Health England, PHE); WHO, World Health Organization

Figure 2 depicts the distribution of patients among the different datasets and how the different case definitions overlap at identifying patients. As seen in panel A, 191,294 patients were present in all datasets, whereas as seen in panel B, that 108,407 patients met all four case definitions. The proportion of patients meeting the case definitions ranged from 56.8% (WHO) to 82.3% (CDC) and varied by geographical region. East Asia and Pacific presented the lowest percentages (33% to 54%, with WHO the lowest and ECDC the highest). The highest percentages were observed for Europe and Central Asia (59% to 84%, with WHO the lowest and CDC the highest). The proportion of patients meeting the case definitions also varied by age. Overall, the age curve followed a bell‐shape pattern (Figure 3A), with the lowest proportion of patients meeting definitions at the extremes of the age distribution (except under 10s). However, the same pattern was not evident when assessing this relationship stratified by region. The bell‐shape pattern applies to Europe and Central Asia but differs in the two other geographical regions. A consistently high number of patients met the case definition across all age groups in South Asia, except for the WHO definition.

**FIGURE 4:**
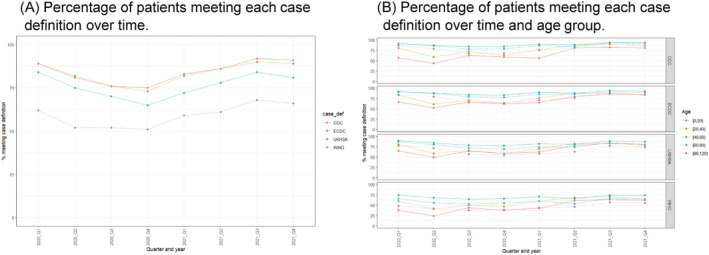
Percentage of patients in the ISARIC database meeting the United States CDC (N = 196,437), ECDC (N = 222,052), UKHSA (N = 218,274) and WHO (N = 209,615) case definitions over time. Panel (A), patients by time. Panel (B), patients by age and time. CDC, Centers for Disease Control, United States; ECDC, European Centre for Disease Prevention and Control; UKHSA, UK Health Security Agency (formerly Public Health England, PHE); WHO, World Health Organization

Temporal variations were observed for all case definitions (Figure 4A), with all case definitions displaying a U‐shaped pattern. Although WHO's remained with a low percentage of patients meeting the case definitions between quarters 2 and 4, 2020, the nadir was observed for all case definitions in quarter 4 (Figure 4A). For all case definitions, progressively higher percentage of patients meeting the case definition increased throughout 2021, reaching a stable point by the end of the year (Figure 4A).

Age variability was primarily present during the first year and progressively decreased over time; most age categories presented a relatively similar percentage of patients meeting the case definitions during the last three quarters of 2021 (Figure 4B). For example, for CDC, the between‐age group differences were close to 30% at the beginning of the pandemic, whereas this was close to 10% during the last quarter of 2022. In addition, although male patients met case definitions more commonly than females, this difference was minimal—the largest observed difference was 4.8 percentual points for WHO's.

### 3.3 CFRs and symptoms

Depending on the case definition dataset (CDC, ECDC, UKHSA and WHO), between 87% and 88% of the study population had a recorded outcome (death or discharge). Model‐adjusted CFRs were similar across case definitions for those who met the criteria but not among those who did not (Table 1). Among those who did not meet the case definitions, variations were between 18.2% (US‐CDC) and 23.1% (WHO), increasing with the proportion of patients not meeting the criteria (Table 1).

**TABLE 1:** Case fatality ratio among cases that met versus those who did not meet each case definition, adjusted for time (by quarter and year), age (in 10‐year groups), sex and region (according to the World Bank classification)

Case definition	Meeting		Not meeting	
	% met	CFR (95% CI)	% not meeting	CFR (95% CI)
CDC	82.8%	25.75 (24.98–26.53)	17.2%	18.2 (17.47–18.92)
ECDC	81.9%	26.8 (26.07–27.54)	18.1%	19.12 (18.43–19.81)
UKHSA	74.6%	26.15 (25.41–26.89)	25.4%	22.21 (21.49–22.94)
WHO	57.3%	26.63 (25.85–27.4)	42.7%	23.09 (22.38–23.81)

Abbreviations: CDC, Centers for Disease Control, United States; CFR, case fatality ratio; CI, confidence interval; ECDC, European Centre for Disease Prevention and Control; UKHSA, UK Health Security Agency (formerly Public Health England, PHE); WHO, World Health Organization.

Patients' symptoms and comorbidities for those who met and did not meet the case definitions are presented in Appendix S2. Overall, the three most common symptoms among those who did not meet the case definitions were shortness of breath, cough and confusion for WHO; confusion, dehydration and fatigue for ECDC; fever, dehydration and confusion for CDC; and shortness of breath, confusion and fatigue for UKHSA. Notably, when assessing the case definition with the lowest number of patients meeting their case definition (WHO's), 34.2% presented with shortness of breath, 15.5% with a history of fever, 15.5% with fatigue and 24.3% with cough. These results indicate that many patients with single symptoms might not meet the case definition for criteria that require a combination of symptoms rather than a fixed number of them (AND vs. OR). In contrast to symptoms, comorbidities were relatively similar across those who met and those who did not meet the case definitions, except for obesity (Appendix S2).

## 4 CONCLUSIONS

### 4.1 Key results

Our analysis of the global ISARIC dataset shows that the clinical presentation of COVID‐19 in hospitalised patients changes across time and geographic regions and that performance of case definitions may be improved by adapting to these variations. Although our findings that patients' age and comorbidities influence presenting symptoms are consistent with other research,^9^, ^10^ it remains concerning that case definitions are least able to identify patients who are most vulnerable to severe outcomes. These patients might risk receiving delayed medical care, or inadvertent cohorting with patients who do not have the disease (including in settings such as nursing homes) and risk further transmission. Our key finding is that the stage of the pandemic and its evolution across geographical regions influences clinical presentation. Similarly, case definitions with complex rules (e.g., AND vs. OR) might not identify patients with common symptoms like cough, fever and shortness of breath.

Our temporal analysis found a decrease in patients meeting the clinical criteria of the case definitions from late 2020 to early 2021. Evolving patient symptoms over time could be caused by changes in circulating variants, varying incidence of co‐infections or varying presentations in different age groups whose infection rates also vary over time. Even though ISARIC aims at enrolling patients hospitalised due to COVID‐19, site‐specific changes to recruitment or data collection practices may also a have significant impact.

For example, a potential confounder is that the decrease in patients meeting the case definitions correlated with a surge in cases in the countries that contributed most heavily to our analysis. When COVID‐19 incidence or test availability is high, testing protocols may be more liberal, and more patients with atypical or asymptomatic disease may be captured (e.g., a patient involved in a road accident is admitted and found to have incidental COVID‐19). On the other hand, admission policies may lead to only severe patients being admitted when the transmission is high. The decision to admit patients may also vary depending on what level of care is available at the time. It is less likely that the patterns observed during 2020 could be due to vaccine uptake; few of the patients in our analyses will have had access to vaccination during late 2020.

The observed differences in the proportion of patients meeting case definitions across regions might reflect population‐level differences in symptom profiles. Alternative explanations include cultural reticence in reporting symptoms, varying criteria for hospital admission (such as countries that used hospitals as isolation facilities for patients with milder disease during early 2020), time from symptom onset to admission (in shorter times, patients will report fewer symptoms^11^), whether patients with milder disease had access to community‐based or alternative services or the number and nature of the patients contributing to data from the different regions. In addition, how clinical case definitions vary by geographical region suggests that their adoption by governments and public health institutions in regions other than those from where they were generated (for ECDC, PHE and CDC) might require adaptation to the local context.

The objective was to evaluate if there were temporal and geographical differences in the proportion of hospitalised COVID‐19 patients meeting frequently used COVID‐19 case definitions, and our findings suggest that to meet the needs of hospital systems, frequent validation against comprehensive natural history data should occur to account for these differences. Furthermore, these definitions should better meet the needs of patients most vulnerable to death from the disease. However, a difficulty with making frequent updates or increasing their complexity to improve their accuracy (e.g., by introducing age‐specific definitions) is that definitions become unwieldy to use—while the actual benefit of the tool is often in being a fast aid to decision‐making. Therefore, epidemiologists and social scientists must balance statistical accuracy with operational efficacy.

In the case of emerging infections, continued revisions of case definitions need to be carried out, as many countries with the highest incidence of emerging infections rely upon international definitions for suspected cases and test eligibility.^12^ One solution may be to advocate for local adaptations of case definitions (WHO endorses this^2^). For example, in the case of COVID‐19, 56% of the 25 countries that account for ~85% of global cases have added lack of taste or smell to their suspected case definition.^12^ Although local adaptations are necessary, this also means that the denominator used when comparing regions would be different. For example, an analysis of different versions of the case definition issued by the National Health Commission in China early during the pandemic estimated that the proportion of detected cases would have increased by over sevenfold if the final version had been implemented earlier.^13^

Presently, some organisations are using the same case definitions for public health surveillance and clinical use, but others (such as CDC) emphasise that their surveillance definitions should not be used for clinical diagnosis. Further research is required to determine if splitting these definitions to calibrate their specific needs is worth the additional costs, resources and complexity, or whether the clinical utility can be enhanced in other ways that do not detract from use in surveillance activities. There are promising reports of artificial intelligence‐driven COVID‐19 triage; however, the implementation of such technology in regions without electronic healthcare systems or effective data sharing frameworks will be difficult.^14^

As a general epidemiological principle, higher detection rates (increased sensitivity) will come at the expense of lower specificity, a well‐known dilemma for diagnostic tests.^15^ However, the decision to modify such cut‐off points at a local level must be weighted based on disease incidence, positive/negative predicted values and the relative cost of a false clinical‐diagnosis result. In a high‐incidence situation, a case definition with high sensitivity (and therefore a high positive predicted value) may positively affect a hospital triage system, as non‐suspected patients will be isolated from suspected patients, and COVID‐19 patients will receive early treatment. Yet, in a low‐incidence scenario, the cost of reducing specificity might lead to a large number of non‐COVID‐19 patients being isolated with COVID‐19 patients while awaiting diagnosis, risking nosocomial transmission. To fully understand these patterns, test‐negative studies (i.e., including non‐COVID‐19 patients) must be conducted to evaluate decision‐making processes while weighing the probabilities of consequences of our public health guidelines.

### 4.2 Strengths and limitations

Our analysis has significant strengths; notably, we use the world's largest international dataset on hospitalised COVID‐19 patients. In addition, we included definitions from multiple leading health agencies, including those globally (WHO) and regionally (ECDC) implemented. We decided not to restrict our analysis to a single organisation's case definition to focus on general advocacy for improved definitions rather than auditing the performance of an organisation. Several limitations were also present. First, our study is focused on hospitalised patients; however, it is expectable that if these patients do not meet the symptom‐based definitions, community cases would not meet them either. Second, variation in data collection methods between countries (electronic health records vs. questionnaire based), our ability to assess only the symptom component of these definitions and the subjective nature of symptoms themselves could have impacted our analysis. To account for this, the ISARIC COVID‐19 platform was launched with a standardised questionnaire. Third, although a large proportion of patients were excluded from the analyses due to incomplete data, we do not expect a selection bias, as most countries were represented in both the initial and the final database. And fourth, although it is well known that COVID‐19 can often be asymptomatic, which would mean that patients would not meet the case definitions, all of our patients were hospitalised.

In conclusion, early diagnosis of COVID‐19 in hospitals is essential to limit the nosocomial spread of disease and provide early, adequate case management to patients, especially where diagnostic capacity is low. Common case definitions for COVID‐19 vary by geographical region and time. We expect geographical and temporal changes to continue due to variations in population structures in different geographic regions, hospital practices, differential vaccine uptake and new variants. Therefore, clinical case definitions should be frequently interrogated to reflect clinical reality.

## FUNDING INFORMATION

This work was made possible by the UK Foreign, Commonwealth and Development Office; Wellcome Trust (215091/Z/18/Z, 205228/Z/16/Z, 220757/Z/20/Z); Bill and Melinda Gates Foundation (OPP1209135); the philanthropic support of the donors to the University of Oxford's COVID‐19 Research Response Fund (0009109); CIHR Coronavirus Rapid Research Funding Opportunity OV2170359 and the co‐ordination in Canada by Sunnybrook Research Institute; endorsement of the Irish Critical Care‐Clinical Trials Group, co‐ordination in Ireland by the Irish Critical Care‐Clinical Trials Network at University College Dublin and funding by the Health Research Board of Ireland (CTN‐2014‐12); the Rapid European COVID‐19 Emergency Response research (RECOVER) (H2020 project 101003589) and European Clinical Research Alliance on Infectious Diseases (ECRAID) (965313); the COVID Clinical Management Team, AIIMS, Rishikesh, India; the COVID‐19 Clinical Management Team, Manipal Hospital Whitefield, Bengaluru, India; Cambridge NIHR Biomedical Research Centre; the dedication and hard work of the Groote Schuur Hospital Covid ICU Team; the Liverpool School of Tropical Medicine and the University of Oxford; the dedication and hard work of the Norwegian SARS‐CoV‐2 Study Team; the Research Council of Norway Grant No. 312780 and a philanthropic donation from Vivaldi Invest A/S owned by Jon Stephenson von Tetzchner; Imperial NIHR Biomedical Research Centre; the Comprehensive Local Research Networks (CLRNs) of which PJMO is an NIHR Senior Investigator (NIHR201385); Innovative Medicines Initiative Joint Undertaking under Grant Agreement No. 115523 COMBACTE, resources of which are composed of financial contribution from the European Union's Seventh Framework Programme (FP7/2007‐2013) and EFPIA companies, in‐kind contribution; the French COVID cohort (NCT04262921) is sponsored by INSERM and is funded by the REACTing (REsearch & ACtion emergING infectious diseases) Consortium and by a grant of the French Ministry of Health (PHRC No. 20‐0424); Stiftungsfonds zur Förderung der Bekämpfung der Tuberkulose und anderer Lungenkrankheiten of the City of Vienna, Project Number: APCOV22BGM; Italian Ministry of Health ‘Fondi Ricerca corrente–L1P6’ to IRCCS Ospedale Sacro Cuore–Don Calabria; Australian Department of Health grant (3273191); Gender Equity Strategic Fund at University of Queensland, Artificial Intelligence for Pandemics (A14PAN) at University of Queensland, the Australian Research Council Centre of Excellence for Engineered Quantum Systems (EQUS, CE170100009) and the Prince Charles Hospital Foundation, Australia; grants from Instituto de Salud Carlos III, Ministerio de Ciencia, Spain; Brazil, National Council for Scientific and Technological Development Scholarship Number 303953/2018‐7; the Firland Foundation, Shoreline, Washington, USA; a grant from foundation Bevordering Onderzoek Franciscus; the South Eastern Norway Health Authority and the Research Council of Norway; Institute for Clinical Research (ICR), National Institutes of Health (NIH) supported by the Ministry of Health Malaysia; and preparedness work conducted by the Short Period Incidence Study of Severe Acute Respiratory Infection.

This work uses data provided by patients and collected by the NHS as part of their care and support #DataSavesLives. The data used for this research were obtained from ISARIC4C. We are extremely grateful to the 2648 frontline NHS clinical and research staff and volunteer medical students who collected these data in challenging circumstances and the generosity of the patients and their families for their individual contributions in these difficult times. The COVID‐19 Clinical Information Network (CO‐CIN) data were collated by ISARIC4C Investigators. Data and Material provision was supported by grants from the National Institute for Health Research (NIHR; Award CO‐CIN‐01), the Medical Research Council (MRC; Grant MC_PC_19059) and the NIHR Health Protection Research Unit (HPRU) in Emerging and Zoonotic Infections at University of Liverpool in partnership with Public Health England (PHE) (Award 200907), NIHR HPRU in Respiratory Infections at Imperial College London with PHE (Award 200927), Liverpool Experimental Cancer Medicine Centre (Grant C18616/A25153), NIHR Biomedical Research Centre at Imperial College London (Award ISBRC‐1215‐20013) and NIHR Clinical Research Network providing infrastructure support. We also acknowledge the support of Jeremy J. Farrar and Nahoko Shindo.

## CONFLICTS OF INTEREST

Allavena, C. declares personal fees from ViiV Healthcare, MSD, Janssen and Gilead, all outside the submitted work. Andréjak, C. declares personal fees for lecture from Astra Zeneca, outside the submitted work. Antonelli, M. declares unrestricted research grants from GE and Estor/Toray and Board participation from Pfizer and Shionogi. All unrelated to the present work. Borie, R. declares personal fees for lecture from Roche, Sanofi and Boehringer Ingelheim, outside the submitted work. Bosse, Hans Martin is co‐investigator for placebo studies in infants and children in clinical trials by Actelion/Janssen (Johnson & Johnson), outside the submitted work. Cheng, M. declares grants from McGill Interdisciplinary Initiative in Infection and Immunity and grants from Canadian Institutes of Health Research, during the conduct of the study; and personal fees from GEn1E Lifesciences (as a member of the scientific advisory board) and personal fees from nplex biosciences (as a member of the scientific advisory board), outside the submitted work. He is the co‐founder of Kanvas Biosciences and owns equity in the company. In addition, Cheng, M. reports a patent Methods for detecting tissue damage, graft versus host disease and infections using cell‐free DNA profiling pending, and a patent Methods for assessing the severity and progression of SARS‐CoV‐2 infections using cell‐free DNA pending. Cholley, B. declares personal fees (for lectures and participation to advisory boards) from Edwards, Amomed, Nordic Pharma and Orion Pharma. Claure‐Del Granado, R. declares personal fees (for lectures and participation to advisory boards) from Nova Biomedical and Medtronic, all outside the submitted work. Cruz‐Bermúdez, J. L. declares personal fees from Elsevier for advice, outside the submitted work. Cummings, M. and O'Donnell, M. participated as investigators for clinical trials evaluating the efficacy and safety of remdesivir (sponsored by Gilead Sciences) and convalescent plasma (sponsored by Amazon) in hospitalised patients with COVID‐19. Support for this work is paid to Columbia University. Dalton, H. declares personal fees for medical director of Innovative ECMO Concepts and honorarium from Abiomed/BREETHE Oxi‐1 and Instrumentation Labs and consultant fee from Entegrion Inc., Medtronic and Hemocue. Dyrhol‐Riise, A. M. declares grants from Gilead outside this work. Deplanque, D. declares personal fees from Biocodex, Bristol‐Myers Squibb and Pfizer (advisory boards). Donnelly, C. A. declares research funding from the UK Medical Research Council and the UK National Institute for Health Research. Douglas, J. J. declares personal fees from lectures from Sunovion and Merck and consulting fees from Pfizer. Durante‐Mangoni, E. declares funding via his institution from MSD and Pfizer and personal fees or participation in advisory boards or participation to the speaker's bureau of Roche, Pfizer, MSD, Angelini, Correvio, Nordic Pharma, Bio‐Merieux, Abbvie, Sanofi‐Aventis, Medtronic, Tyrx and DiaSorin. Grasselli, G. declares personal fees from Getinge, Biotest, Draeger Medical, Fisher & Paykel and MSD and unrestricted research grant from MSD and Fisher & Paykel, all outside the submitted work. Gruner, H. declares grants from Bayer outside the submitted work. Guerguerian, A. M. participated as site investigator for the Hospital For Sick Children, Toronto, Canada, as a site through SPRINT‐SARI Study via the Canadian Critical Care Trials Group sponsored in part by the Canadian Institutes of Health Research. Hammond, T. C. declares consulting fees from Regeneron, Pfizer and Agenus. Ho, A. declares grant funding from Medical Research Council UK, Scottish Funding Council ‐ Grand Challenges Research Fund and the Wellcome Trust, outside this submitted work. Holter, J. C. reports grants from Research Council of Norway Grant No. 312780 and from Vivaldi Invest A/S owned by Jon Stephenson von Tetzchner, during the conduct of the study. Hulot, J. S. reports grants from Bioserenity, Sanofi, Servier and Novo Nordisk and speaker, advisory board or consultancy fees from Amgen, Astra Zeneca, Bayer, Bioserenity, Boerhinger Ingelheim, Bristol‐Myers Squibb, MSD, Novartis, Novo Nordisk and Vifor (all unrelated to the present work). Kalleberg, K. T. is a founder and shareholder of the company Age Labs, which develops epigenetic tests, including one for COVID‐19 severity. Kimmoun, A. declares personal fees (payment for lectures) from Baxter, Aguettant and Aspen. Kumar, D. declares grants and personal fees from Roche, GSK and Merck and personal fees from Pfizer and Sanofi. Kutsogiannis, D. J. declares personal fees for a lecture from Tabuk Pharmaceuticals and the Saudi Critical Care Society. Kutsyna, G. declares the study consulting fee for clinical trial ClinicalTrials.gov Identifier: NCT04762628. Laffey, J. reports that he has received fees for consultancy from GlaxoSmithKline and from Baxter Therapeutics for work outside the scope of this work. Lairez, O. declares grant funding from Pfizer; conference fees from Amicus, GE Healthcare, Novartis, Sanofi‐Genzyme and Takeda‐Shire; and consultancy fees from Alnylam, Amicus, Pfizer and Takeda‐Shire. Lee, J. reports grants from European Commission PREPARE Grant Agreement No. 602525, European Commission RECOVER Grant Agreement No. 101003589 and European Commission ECRAID Grant Agreement 965313 supporting the conduct, co‐ordination and management of the work. Lee, T. C. declares research salary support from les Fonds de recherche du Québec – Santé. Lefèvre, B. declares travel/accommodation/meeting expenses from Mylan and Gilead, all outside the submitted work. Lellouche, F. declares grants from CIHR for COVID‐19 studies, is co‐founder and administrator of Oxynov.inc and declares fees from Fisher & Paykel, Vygon and Novus. Lemaignen, A. declares personal fees (payment for lectures) from MSD and Gilead and travel/accommodation/meeting expenses from Pfizer. Leone, M. declares personal fees from Gilead, MSD, Aspen, Ambu and Amomed. Lescure, F. X. declares personal fees (payment for lectures) from Gilead and MSD and travel/accommodation/meeting expenses from Astellas, Eumedica and MSD. Lim, W. S. declares his institution has received unrestricted investigator‐initiated research funding from Pfizer for an unrelated multicentre cohort study in which he is the Chief Investigator and research funding from the National Institute for Health Research, UK, for various clinical trials outside the submitted work. Liu, K. reports personal fees from MERA and receives a salary from TXP Medical completely outside the submitted work. Maier, Lars S. has nothing to declare with respect to the present work. Martin‐Blondel, G. declares support for attending meetings and personal fees from BMS, MSD, Janssen, Sanofi, Pfizer and Gilead for lectures outside the submitted work. Martin‐Loeches, I. declares lectures for Gilead, Thermofisher, Pfizer and MSD; advisory board participation for Fresenius Kabi, Advanz Pharma, Gilead, Accelerate and Merck; and consulting fees for Gilead outside of the submitted work. Martín‐Quiros, A. declares consulting fees for Gilead. Mentré, F. declares consulting fees from IPSEN, Servier and Da Volterra and reports research grants to her group from Sanofi, Roche, Servier and Da Voleterra, all outside the submitted work. Montrucchio, G. declares personal fees for lecture from Pfizer and Gilead outside the submitted work. Murthy, S. declares receiving salary support from the Health Research Foundation and Innovative Medicines Canada Chair in Pandemic Preparedness Research. Nichol, A. declares a grant from the Health Research Board of Ireland to support data collection in Ireland (CTN‐2014‐012), an unrestricted grant from BAXTER for the TAME trial kidney substudy and consultancy fees paid to his institution from AM‐PHARMA. Nseir, S. declares lectures for Gilead, Pfizer, MSD, Biomérieux, Fisher & Paykel and Bio Rad, outside the submitted work. Openshaw, P. has served on scientific advisory boards for Janssen/J&J, Oxford Immunotech Ltd, GSK, Nestle and Pfizer (fees to Imperial College). He is Imperial College lead investigator on EMINENT, a consortium funded by the MRC and GSK. He is a member of the RSV Consortium in Europe (RESCEU) and Inno4Vac, Innovative Medicines Initiatives (IMI) from the European Union. Peltan, I. D. declares grant support from the National Institutes of Health and, outside the submitted work, grant support from Centers for Disease Control and Prevention, National Institutes of Health, and Jannsen and payments to his institution from Regeneron and Asahi Kasei Pharma. Pesenti, A. declares personal fees from Maquet, Novalung/Xenios, Baxter and Boehringer Ingelheim. Peytavin, G. declares consulting fees (for lectures and/or participation in advisory boards) and travel grants from Gilead Sciences, Janssen, Merck, Takeda, Theratechnologies and ViiV Healthcare. Poissy, J. declares personal fees from Gilead for lectures, outside the submitting work. Povoa, P. declares personal fees (for lectures and advisory boards) from MSD, Technophage, Sanofi and Gilead. Póvoas, D. declares consulting fees (for lectures and/or participation in advisory boards) from Roche and ViiV Healthcare and travel/accommodation/meeting expenses from Abbvie, Gilead Sciences, Janssen Cilag, Merck Sharp & Dohme and ViiV Healthcare. Rewa, O. declares honoraria from Baxter Healthcare Inc and Leading Biosciences Inc. Rossanese, A. declares consulting fees (for lectures and/or participation to advisory boards) from Emergent BioSolutions and Sanofi Pasteur, but all outside of the frame of the submitted work. Săndulescu, O. has been an investigator in COVID‐19 clinical trials by Algernon Pharmaceuticals, Atea Pharmaceuticals, Regeneron Pharmaceuticals, Diffusion Pharmaceuticals, Celltrion, Inc. and Atriva Therapeutics, outside the scope of the submitted work. Semple, M. G. reports grants from DHSC National Institute of Health Research UK, from the Medical Research Council UK and from the Health Protection Research Unit in Emerging & Zoonotic Infections, University of Liverpool, supporting the conduct of the study; other interest in Integrum Scientific LLC, Greensboro, NC, USA, outside the submitted work. Serpa Neto, A. declares personal lecture fees from Drager outside the submitted work. Serrano‐Balazote, P. declares funding via his institution from Novartis and Janssen and personal fees or participation in advisory boards or participation to the speaker's bureau of Roche, all outside of the submitted work. Shrapnel, S. participated as an investigator for an observational study analysing ICU patients with COVID‐19 (for the Critical Care Consortium including ECMOCARD) funded by The Prince Charles Hospital Foundation during the conduct of this study. Streinu‐Cercel, Anca has been an investigator in COVID‐19 clinical trials by Algernon Pharmaceuticals, Atea Pharmaceuticals, Regeneron Pharmaceuticals, Diffusion Pharmaceuticals, Celltrion, Inc. and Atriva Therapeutics, outside the scope of the submitted work. Summers, C. reports that she has received fees for consultancy for Abbvie and Roche relating to COVID‐19 therapeutics. She was also the UK Chief Investigator of a GlaxoSmithKline plc sponsored study of a therapy for COVID and is a member of the UK COVID Therapeutic Advisory Panel (UK‐CTAP). Outside the scope of this work, Dr Summers' institution receives research grants from the Wellcome Trust, UKRI/MRC, National Institute for Health Research (NIHR), GlaxoSmithKline and AstraZeneca to support research in her laboratory. Susanne Dudman reports grants from Research Council of Norway Grant No. 312780. Tedder, R. reports grants from MRC/UKRI during the conduct of the study. In addition, R. Tedder has a patent United Kingdom Patent Application No. 2014047.1 ‘SARS‐CoV‐2 antibody detection assay’ issued. Terzi, N. reports personal fees from Pfizer, outside the submitted work. Timsit, J. F. participated in an advisory board for MSD, Pfizer, nabriva, Gilead, Shionoghi and Medimune outside the submitted work and declared lecture fees from MSD, Biomerieux, Pfizer and Shionoghi. Turtle, L. reports grants from MRC/UKRI during the conduct of the study and fees from Eisai for delivering a lecture related to COVID‐19 and cancer, paid to the University of Liverpool. Ullrich, R. reports grant funding to his institution from Apeptico, APEIRON, Biotest, Bayer, CCORE and Philips, as well as personal fees from Biotest. He holds European patent EP15189777.4 ‘Blood purification device’ and equity in CCORE Technology GesmbH, a medical device research and development company. Visseaux, B. declares personal fees from BioMérieux, Qiagen and Gilead and research grants from Qiagen, all outside the submitted work. West, E. reports grant funding from the Firland Foundation, the US‐CDC and the Bill and Melinda Gates Foundation for studies of COVID‐19 and grant funding from the US NIH for studies of other respiratory infections. Søraas, A. is a founder of the company Age Labs, which develops epigenetic tests including for COVID‐19 severity.

## ETHICS

The ISARIC‐WHO Clinical Characterisation protocol was approved by the World Health Organization Ethics Review Committee (RPC571 and RPC572). Local ethics approval was obtained for each participating country and site according to local requirements. Informed consent practices were implemented in each site according to the requirements approved by the local ethics committee.

## AUTHOR CONTRIBUTIONS

**ISARIC Clinical Characterisation Group**: Writing – review and editing (equal). **Joaquin Baruch**: Conceptualisation (lead); data curation (supporting); formal analysis (lead); investigation (equal); methodology (equal); validation (equal); visualisation (equal); writing – original draft (lead); writing – review and editing (lead). **Amanda Rojek**: Conceptualisation (equal); investigation (equal); methodology (equal); writing – original draft (lead); writing – review and editing (lead). **Christiana Kartsonaki**: Conceptualisation (equal); formal analysis (supporting); methodology (supporting); visualisation (supporting); writing – review and editing (equal). **Bharath K. T. Vijayaraghavan**: Conceptualisation (equal); methodology (equal); writing – original draft (equal); writing – review and editing (equal). **Bronner P. Gonçalves**: Conceptualisation (equal); investigation (equal); methodology (equal); visualisation (equal); writing – original draft (equal); writing – review and editing (equal). **Mark G. Pritchard**: Conceptualisation (equal); methodology (equal); writing – original draft (equal); writing – review and editing (equal). **Laura Merson**: Funding acquisition (equal); investigation (equal); methodology (equal); project administration (equal); writing – original draft (equal); writing – review and editing (equal). **Jake Dunning**: Funding acquisition (equal); writing – original draft (equal); writing – review and editing (equal). **Matthew Hall**: Conceptualisation (equal); writing – review and editing (equal). **Louise Sigfrid**: Conceptualisation (equal); writing – review and editing (equal). **Barbara W. Citarella**: Data curation (equal); methodology (equal); writing – review and editing (equal). **Srinivas Murthy**: Methodology (equal); writing – review and editing (equal). **Trokon O. Yeabah**: Writing – review and editing (equal). **Piero Olliaro**: Conceptualisation (equal); funding acquisition (equal); investigation (equal); methodology (equal); project administration (equal); supervision (equal); writing – original draft (equal); writing – review and editing (equal).

### PEER REVIEW

The peer review history for this article is available at https://publons.com/publon/10.1111/irv.13039.

## Supporting information


**Appendix S1.** Case definitions used and rules applied.

**Appendix S2.** Symptoms and comorbidities by patients that did not meet the UKHSA, CDC ‐ United states, ECDC, and WHO COVID‐19 case definitions.

## Data Availability

The majority of this database is available to external researchers via application to our Data Access Committee at https://www.iddo.org/covid19/data-sharing/accessing-data.

## References

[1] CDC . Coronavirus Disease 2019 (COVID‐19) 2020 Interim Case Definition. 2021. Available at: https://ndc.services.cdc.gov/case-definitions/coronavirus-disease-2019-2021/

[2] ECDC . Case definition for coronavirus disease 2019 (COVID‐19), as of 3 December 2020. 2020. Available at: https://www.ecdc.europa.eu/en/covid-19/surveillance/case-definition

[3] UKHSA . COVID‐19: investigation and initial clinical management of possible cases. 2020. Available at: https://www.gov.uk/government/publications/wuhan-novel-coronavirus-initial-investigation-of-possible-cases/investigation-and-initial-clinical-management-of-possible-cases-of-wuhan-novel-coronavirus-wn-cov-infection

[4] WHO . World Health Organization. WHO COVID‐19: case definition. Updated in public health surveillance for COVID‐19. 2020. Available at: https://www.who.int/publications/i/item/WHO-2019-nCoV-Surveillance_Case_Definition-2020.2

[5] WHO . Clinical management living guidance COVID‐19. 2021: 16–44. Available at: https://apps.who.int/iris/handle/10665/338882

[6] World Health Organization . A clinical case definition of post COVID‐19 condition by a Delphi consensus. Who 2020.

[7] ISARIC CCG . The value of open‐source clinical science in pandemic response: lessons from ISARIC. Lancet. 2021;19‐21. 10.1016/S1473-3099(21)00565-X PMC8489876 34619109

[8] Muller CJ , Maclehose RF . Estimating predicted probabilities from logistic regression: different methods correspond to different target populations. Int J Epidemiol. 2014;43(3):962‐970. doi:10.1093/ije/dyu029 24603316 PMC4052139

[9] Docherty AB , Harrison EM , Green CA , et al. Features of 20 133 UK patients in hospital with covid‐19 using the ISARIC WHO Clinical Characterisation Protocol: prospective observational cohort study. BMJ. 2020;369:1‐12. doi:10.1136/bmj.m1985 PMC7243036 32444460

[10] ISARIC CCG . COVID‐19 symptoms at hospital admission vary with age and sex: results from the ISARIC prospective multinational observational study. Infection. 2021;49(5):889‐905. doi:10.1007/s15010-021-01599-5 34170486 PMC8231091

[11] Hall M , Baruch J , Carson G , et al. Ten months of temporal variation in the clinical journey of hospitalised patients with COVID‐19: an observational cohort. Elife. 2021;10:1‐30. 10.7554/eLife.70970 PMC8791638 34812731

[12] Suthar AB , Schubert S , Garon J , Couture A , Brown AM , Charania S . Coronavirus disease case definitions, diagnostic testing criteria, and surveillance in 25 countries with highest reported case counts. Emerg Infect Dis. 2022;28(1):148‐156. doi:10.3201/eid2801.211082 34932450 PMC8714223

[13] Tsang TK , Wu P , Lin Y , Lau EHY , Leung GM , Cowling BJ . Effect of changing case definitions for COVID‐19 on the epidemic curve and transmission parameters in mainland China: a modelling study. Lancet Public Health. 2020;5(5):e289‐e296. doi:10.1016/S2468-2667(20)30089-X 32330458 PMC7173814

[14] Soltan AAS , Yang J , Pattanshetty R , et al. Real‐world evaluation of rapid and laboratory‐free COVID‐19 triage for emergency care: external validation and pilot deployment of artificial intelligence driven screening. Lancet Digit Heal. 2022;4(4):e266‐e278. doi:10.1016/S2589-7500(21)00272-7 PMC8906813 35279399

[15] Peeling RW , Olliaro PL , Boeras DI , Fongwen N . Scaling up COVID‐19 rapid antigen tests: promises and challenges. Lancet Infect Dis. 2021;21(9):e290‐e295. doi:10.1016/S1473-3099(21)00048-7 33636148 PMC7906660

