# Supplementary material for: Symptom‐based case definitions for COVID‐19: Time and geographical variations for detection at hospital admission among 260,000 patients
Source: Influenza Other Respir Viruses. 2022 Sep 5;16(6):1040–50. doi: 10.1111/irv.13039 (PMC9530510; doi:10.1111/irv.13039)
Supplement: Supplementary file 1 — Appendix S1. Case definitions used and rules applied. [file IRV-16-1040-s002.docx]

**Appendix 1.** Case definitions used and rules applied.

The following assumptions and exclusions were followed for the case definitions that are based on the presence of one of a number of symptoms (PHE and ECDC):

1. Patients with at least one of the symptoms were considered to have met the case definition–even if other symptoms were missing.
2. Patients with “no” on all the symptoms required were considered as not meeting the case definition.
3. Patients with some symptoms as “no” but at least one of them missing were excluded from the analysis, given that we cannot know if all symptoms were truly absent.

In contrast, we used the following exclusions for the case definitions based on at least a certain number (*n*) of symptoms from a list of symptoms (WHO and CDC):

1. Patients with more than *n* symptoms reported were considered as meeting the case definition (even if some symptoms were missing),
2. Patients with fewer than *n* symptoms reported and no other missing symptom were considered as not meeting the case definition,
3. Patients with fewer than *n* symptoms missing and all other symptoms marked as absent were considered as not meeting the case definition, and
4. Patients with more missing symptoms than *n* were removed from the dataset for that case definition, given that we cannot assert their true status.

**Change in case definitions over time for CDC**

| **CDC** | **Date** |
| --- | --- |
| At least two of the following symptoms: fever (measured or subjective), chills (not available), rigors (not available), myalgia, headache, sore throat, new olfactory and taste disorder(s) **OR**  At least one of the following symptoms: cough, shortness of breath, or difficulty breathing (not available) | From January 1^st^ to April 4^th^, 2020  <https://ndc.services.cdc.gov/case-definitions/coronavirus-disease-2019-2020/> |
| At least two of the following symptoms: fever (measured or subjective), chills (not available), rigors (not available), myalgia, headache, sore throat, nausea or vomiting, diarrhea, fatigue, congestion or runny nose. **OR**  Any one of the following symptoms: cough, shortness of breath, difficulty breathing (not available), new olfactory disorder, new taste disorder | From April 5^th^ to August 5^th^, 2020  <https://ndc.services.cdc.gov/case-definitions/coronavirus-disease-2019-2020-08-05/> |
| Acute onset or worsening of at least two of the following symptoms or signs: fever (measured or subjective), chills (not available), rigors (not available), myalgia, headache, sore throat, nausea or vomiting, diarrhea, fatigue, congestion or runny nose. **OR** Acute onset or worsening of any one of the following symptoms or signs: cough, shortness of breath, difficulty breathing (not available), olfactory disorder, taste disorder, confusion or change in mental status, persistent pain or pressure in the chest, pale, gray, or blue-colored skin, lips, or nail beds (not available), depending on skin tone, inability to wake or stay awake (not available). | From August 5^th^ onwards  <https://ndc.services.cdc.gov/case-definitions/coronavirus-disease-2019-2021/> |

Change in case definitions over time for ECDC

| **ECDC** | **Date** |
| --- | --- |
| At least one of the following:  Cough, sore throat, or shortness of breath | January 25^st^ to 24^th^ of February, 2020  Personal communication from ECDC |
| At least one of the following:  Cough, fever, or shortness of breath | February 25^th^ to 1^nd^ of March, 2020  Personal communication from ECDC |
| At least one of the following:  Cough, fever, or shortness of breath | March 2^nd^ to 28^th^ of May, 2020  Personal communication from ECDC |
| At least one of the following:  Cough, fever, shortness of breath, or sudden onset of anosmia, ageusia or dysgeusia | May 29^th^ to 2^nd^ of December, 2020  Personal communication from ECDC |
| At least one of the following symptoms:  cough, fever, shortness of breath, sudden onset of anosmia, ageusia or dysgeusia | Current, 3 December, 2020  <https://www.ecdc.europa.eu/en/covid-19/surveillance/case-definition> |

Change in case definitions over time for WHO

| **WHO** | **Date** |
| --- | --- |
| Generic information: | All WHO case definitions can be found [here](https://apps.who.int/iris/discover?scope=%2F&query=Public+health+surveillance+for+COVID-19%3A+interim+guidance&submit=)  The first 100 records of this page were revised when looking for guidelines |
| Fever AND cough | Jan 11, 2020 |
| Fever AND cough | Jan 15, 2020 |
| Fever AND cough | Jan 21, 2020 |
| Fever AND cough | Jan 31, 2020 |
| Fever AND (Cough or SOB, or other respiratory disease symptom)  For “other” we took:  SOB, cough, runny nose, sore throat | Feb 27, 2020 |
| Fever AND (Cough or SOB, or other respiratory disease symptom)  For “other” we took:  SOB, cough, runny nose, sore throat | March 10, 2020 |
| Fever AND (Cough or SOB, or other respiratory disease symptom)  For “other” we took:  SOB, cough, runny nose, sore throat | March 26, 2020 |
| Acute onset of fever AND cough; **OR** Acute onset of ANY THREE OR MORE of the following signs or symptoms: Fever, cough, general weakness/fatigue1, headache, myalgia, sore throat, coryza, dyspnoea, anorexia/nausea/vomiting1, diarrhoea, altered mental status. | 7 august, 2020 |
| Acute onset of fever AND cough; **OR** Acute onset of ANY THREE OR MORE of the following signs or symptoms: Fever, cough, general weakness/fatigue1, headache, myalgia, sore throat, coryza, dyspnoea, anorexia/nausea/vomiting1, diarrhoea, altered mental status. | Current, 16 dec 2020  <https://www.who.int/publications/i/item/WHO-2019-nCoV-Surveillance_Case_Definition-2020.2> |

Change in case definitions over time for PHE

| **UKHSA** | **Data** |
| --- | --- |
| General information | **Sources of information:**   - Dates of updates and summary of updates (headers for each section below) were identified here: <https://www.gov.uk/government/publications/wuhan-novel-coronavirus-initial-investigation-of-possible-cases#full-publication-update-history> - The current UKHSA ‘possible’ case definition (for clinical purposes) is available here: <https://www.gov.uk/government/publications/wuhan-novel-coronavirus-initial-investigation-of-possible-cases/investigation-and-initial-clinical-management-of-possible-cases-of-wuhan-novel-coronavirus-wn-cov-infection#criteria> - Archived versions of the above webpage, where available and based on the identified dates of updates to guidance, where obtained by performing a search of the above URL on <https://web.archive.org/> - For January 2020, not all guidance and guidance updates was archived on web.archive.org; therefore, CMO letters and flow-charts for medical professionals were reviewed on <https://www.cas.mhra.gov.uk/Help/CoronavirusAlerts.aspx>   The original interim definition for a possible case, 10 January 2020, could not be found online; the information provided |
| Fever or history of fever AND at least one of:  Shortness of breath, cough, or sore throat | January 10th to 14th, 2020 |
| At least one of: shortness of breath, cough, or sore throat | January 15^th^ to January 30^st^, 2020 |
| At least one of fever, shortness of breath, or cough | January 31^st^ to February 5^th^, 2020 |
| Shortness of breath or cough  OR  Fever with no other symptoms* | February 6^th^ to February 10^th^, 2020 |
| Shortness of breath or cough  OR  Fever with no other symptoms* | February 11^th^ to March 9^th^, 2020 |
| Shortness of breath or cough  OR  Fever with no other symptoms* | March 10^th^ to March 11^th^ , 2020 |
| Shortness of breath or cough  OR  Fever with no other symptoms* | March 12^th^ to March 13^th^, 2020 |
| Cough OR fever OR loss/change of smell or taste | March 13^th^ to May 17^th^ |
| Cough OR fever OR loss/change of smell or taste | May 18^th^ to October 1^st^ |
| New continuous cough, **or,** temperature ≥37.8°C**, or,** loss of, or change in, normal sense of smell (anosmia) or taste (ageusia) | Current, 28 sept 2020  <https://www.gov.uk/government/publications/wuhan-novel-coronavirus-initial-investigation-of-possible-cases/investigation-and-initial-clinical-management-of-possible-cases-of-wuhan-novel-coronavirus-wn-cov-infection> |

*Between 6^th^ of February and 13^th^ of March, “fever with no other symptoms” was not evaluated due to the complexity of missing data in this question. For example, all symptoms must be marked as “no” except for fever, and a patient with 1 symptom missing would be treated as missing and excluded from the dataset. Instead, the other case definition “Shortness of breath” or cough was used.
