# Supplementary material for: Symptom‐based case definitions for COVID‐19: Time and geographical variations for detection at hospital admission among 260,000 patients
Source: Influenza Other Respir Viruses. 2022 Sep 5;16(6):1040–50. doi: 10.1111/irv.13039 (PMC9530510; doi:10.1111/irv.13039)
Supplement: Supplementary file 2 — Appendix S2. Symptoms and comorbidities by patients that did not meet the UKHSA, CDC ‐ United states, ECDC, and WHO COVID‐19 case definitions. [file IRV-16-1040-s001.docx]

**Appendix 2**. Symptoms and comorbidities by patients that did not meet the UKHSA, CDC - United states, ECDC, and WHO COVID-19 case definitions.

Symptoms by patients that meet and did not meet the UKHSA case definition

|  | Case definition met | |
| --- | --- | --- |
| Parameter | Yes (%) | No (%) |
| Abdominal pain |  |  |
| No | 129855 (80) | 50840 (90.9) |
| Yes | 12184 (7.5) | 4097 (7.3) |
| Unknown | 20291 (12.5) | 1007 (1.8) |
| Confusion |  |  |
| No | 118110 (72.8) | 42324 (75.7) |
| Yes | 26116 (16.1) | 9381 (16.8) |
| Unknown | 18104 (11.2) | 4239 (7.6) |
| Bleeding |  |  |
| No | 139279 (85.8) | 53501 (95.6) |
| Yes | 2018 (1.2) | 1360 (2.4) |
| Unknown | 21033 (13) | 1083 (1.9) |
| Chest pain |  |  |
| No | 120854 (74.4) | 51893 (92.8) |
| Yes | 23008 (14.2) | 3109 (5.6) |
| Unknown | 18468 (11.4) | 942 (1.7) |
| Conjunctivitis |  |  |
| No | 133511 (82.2) | 54348 (97.1) |
| Yes | 534 (0.3) | 69 (0.1) |
| Unknown | 28285 (17.4) | 1527 (2.7) |
| Cough |  |  |
| No | 32128 (19.8) | 55927 (100) |
| Yes | 126294 (77.8) | 17 (0) |
| Unknown | 3908 (2.4) |  |
| Diarrhoea |  |  |
| No | 118981 (73.3) | 51435 (91.9) |
| Yes | 27323 (16.8) | 3747 (6.7) |
| Unknown | 16026 (9.9) | 762 (1.4) |
| Fatigue malaise |  |  |
| No | 76650 (47.2) | 46375 (82.9) |
| Yes | 64072 (39.5) | 8102 (14.5) |
| Unknown | 21608 (13.3) | 1467 (2.6) |
| Headache |  |  |
| No | 116649 (71.9) | 52532 (93.9) |
| Yes | 17587 (10.8) | 1875 (3.4) |
| Unknown | 28094 (17.3) | 1537 (2.7) |
| History of fever |  |  |
| No | 36168 (22.3) | 55498 (99.2) |
| Yes | 121424 (74.8) | 431 (0.8) |
| Unknown | 4738 (2.9) | 15 (0) |
| lost altered sense of smell |  |  |
| No | 100684 (62) | 50922 (91) |
| Yes | 10630 (6.5) | 148 (0.3) |
| Unknown | 51016 (31.4) | 4874 (8.7) |
| Lost altered sense of taste |  |  |
| No | 95756 (59) | 50753 (90.7) |
| Yes | 12609 (7.8) | 115 (0.2) |
| Unknown | 53965 (33.2) | 5076 (9.1) |
| Muscle aches joint pain |  |  |
| No | 105455 (65) | 51892 (92.8) |
| Yes | 28811 (17.7) | 2497 (4.5) |
| Unknown | 28064 (17.3) | 1555 (2.8) |
| Runny_nose |  |  |
| No | 124219 (76.5) | 53839 (96.2) |
| Yes | 4471 (2.8) | 387 (0.7) |
| Unknown | 33640 (20.7) | 1718 (3.1) |
| Seizures |  |  |
| No | 138512 (85.3) | 50614 (90.5) |
| Yes | 1253 (0.8) | 730 (1.3) |
| Unknown | 22565 (13.9) | 4600 (8.2) |
| Severe dehydration |  |  |
| No | 56736 (35) | 19245 (34.4) |
| Yes | 7921 (4.9) | 2622 (4.7) |
| Unknown | 97673 (60.2) | 34077 (60.9) |
| Shortness of breath |  |  |
| No | 42895 (26.4) | 41193 (73.6) |
| Yes | 114155 (70.3) | 14475 (25.9) |
| Unknown | 5280 (3.3) | 276 (0.5) |
| Skin rash |  |  |
| No | 134586 (82.9) | 53659 (95.9) |
| Yes | 3162 (1.9) | 982 (1.8) |
| Unknown | 24582 (15.1) | 1303 (2.3) |
| Sore throat |  |  |
| No | 118063 (72.7) | 53534 (95.7) |
| Yes | 11360 (7) | 825 (1.5) |
| Unknown | 32907 (20.3) | 1585 (2.8) |
| Nausea |  |  |
| No | 117982 (72.7) | 49573 (88.6) |
| Yes | 27809 (17.1) | 5587 (10) |
| Unknown | 16539 (10.2) | 784 (1.4) |
| Wheezing |  |  |
| No | 126414 (77.9) | 53807 (96.2) |
| Yes | 10309 (6.4) | 901 (1.6) |
| Unknown | 25607 (15.8) | 1236 (2.2) |

Comorbidities by patients that meet and did not meet the UKHSA case definition

|  | Case definition met | |
| --- | --- | --- |
| Parameter | Yes (%) | No (%) |
| AIDS/HIV |  |  |
| No | 142542 (87.8) | 52983 (94.7) |
| Yes | 985 (0.6) | 163 (0.3) |
| Unknown | 18803 (11.6) | 2798 (5) |
| Asthma |  |  |
| No | 128308 (79) | 48834 (87.3) |
| Yes | 21058 (13) | 5106 (9.1) |
| Unknown | 12964 (8) | 2004 (3.6) |
| Chronic cardiac disease |  |  |
| No | 114547 (70.6) | 37825 (67.6) |
| Yes | 38055 (23.4) | 16798 (30) |
| Unknown | 9728 (6) | 1321 (2.4) |
| Chronic kidney disease |  |  |
| No | 129768 (79.9) | 45176 (80.8) |
| Yes | 19922 (12.3) | 8875 (15.9) |
| Unknown | 12640 (7.8) | 1893 (3.4) |
| Chronic neurological disorder |  |  |
| No | 135119 (83.2) | 47198 (84.4) |
| Yes | 14227 (8.8) | 6764 (12.1) |
| Unknown | 12984 (8) | 1982 (3.5) |
| Chronic pulmonary disease |  |  |
| No | 128500 (79.2) | 47753 (85.4) |
| Yes | 23647 (14.6) | 6788 (12.1) |
| Unknown | 10183 (6.3) | 1403 (2.5) |
| Diabetes |  |  |
| No | 109271 (67.3) | 40397 (72.2) |
| Yes | 41010 (25.3) | 13692 (24.5) |
| Unknown | 12049 (7.4) | 1855 (3.3) |
| Hypertension |  |  |
| No | 71803 (44.2) | 28462 (50.9) |
| Yes | 58215 (35.9) | 22393 (40) |
| Unknown | 32312 (19.9) | 5089 (9.1) |
| Liver disease |  |  |
| No | 150322 (92.6) | 52899 (94.6) |
| Yes | 4499 (2.8) | 1993 (3.6) |
| Unknown | 7509 (4.6) | 1052 (1.9) |
| Malignant neoplasm |  |  |
| No | 138216 (85.1) | 48778 (87.2) |
| Yes | 13368 (8.2) | 5715 (10.2) |
| Unknown | 10746 (6.6) | 1451 (2.6) |
| Malnutrition |  |  |
| No | 138934 (85.6) | 49777 (89) |
| Yes | 2414 (1.5) | 1409 (2.5) |
| Unknown | 20982 (12.9) | 4758 (8.5) |
| Obesity |  |  |
| No | 112525 (69.3) | 45197 (80.8) |
| Yes | 22573 (13.9) | 4602 (8.2) |
| Unknown | 27232 (16.8) | 6145 (11) |
| Smoking |  |  |
| No | 53332 (32.9) | 15131 (27) |
| Yes | 38497 (23.7) | 11390 (20.4) |
| Unknown | 70501 (43.4) | 29423 (52.6) |

Comorbidities by patients that meet and did not meet the ECDC case definition

|  | Case definition met | |
| --- | --- | --- |
| Parameter | Yes (%) | No (%) |
| AIDS/HIV |  |  |
| No | 159456 (88) | 38942 (95.2) |
| Yes | 1046 (0.6) | 120 (0.3) |
| Unknown | 20631 (11.4) | 1857 (4.5) |
| Asthma |  |  |
| No | 143628 (79.3) | 36188 (88.4) |
| Yes | 23246 (12.8) | 3387 (8.3) |
| Unknown | 14259 (7.9) | 1344 (3.3) |
| Chronic cardiac disease |  |  |
| No | 125987 (69.6) | 28499 (69.6) |
| Yes | 44592 (24.6) | 11584 (28.3) |
| Unknown | 10554 (5.8) | 836 (2) |
| Chronic kidney disease |  |  |
| No | 143956 (79.5) | 33397 (81.6) |
| Yes | 23313 (12.9) | 6231 (15.2) |
| Unknown | 13864 (7.7) | 1291 (3.2) |
| Chronic neurological disorder |  |  |
| No | 150531 (83.1) | 34464 (84.2) |
| Yes | 16319 (9) | 5087 (12.4) |
| Unknown | 14283 (7.9) | 1368 (3.3) |
| Chronic pulmonary disease |  |  |
| No | 142851 (78.9) | 35948 (87.9) |
| Yes | 27179 (15) | 4065 (9.9) |
| Unknown | 11103 (6.1) | 906 (2.2) |
| Diabetes |  |  |
| No | 121715 (67.2) | 30083 (73.5) |
| Yes | 46249 (25.5) | 9609 (23.5) |
| Unknown | 13169 (7.3) | 1227 (3) |
| Hypertension |  |  |
| No | 79838 (44.1) | 21746 (53.1) |
| Yes | 66394 (36.7) | 15901 (38.9) |
| Unknown | 34901 (19.3) | 3272 (8) |
| Liver disease |  |  |
| No | 167959 (92.7) | 38798 (94.8) |
| Yes | 5138 (2.8) | 1461 (3.6) |
| Unknown | 8036 (4.4) | 660 (1.6) |
| Malignant neoplasm |  |  |
| No | 154176 (85.1) | 35772 (87.4) |
| Yes | 15236 (8.4) | 4208 (10.3) |
| Unknown | 11721 (6.5) | 939 (2.3) |
| Malnutrition |  |  |
| No | 154843 (85.5) | 36452 (89.1) |
| Yes | 2839 (1.6) | 1089 (2.7) |
| Unknown | 23451 (12.9) | 3378 (8.3) |
| Obesity |  |  |
| No | 125974 (69.5) | 33728 (82.4) |
| Yes | 24889 (13.7) | 2866 (7) |
| Unknown | 30270 (16.7) | 4325 (10.6) |
| Smoking |  |  |
| No | 57437 (31.7) | 11755 (28.7) |
| Yes | 42896 (23.7) | 7962 (19.5) |
| Unknown | 80800 (44.6) | 21202 (51.8) |

Symptoms by patients that meet and do not meet the ECDC case definition

|  | Case definition met | |
| --- | --- | --- |
| Parameter | Yes (%) | No (%) |
| Abdominal pain |  |  |
| No | 144755 (79.9) | 37263 (91.1) |
| Yes | 13290 (7.3) | 3225 (7.9) |
| Unknown | 23088 (12.7) | 431 (1.1) |
| Confusion |  |  |
| No | 131120 (72.4) | 30472 (74.5) |
| Yes | 29719 (16.4) | 6637 (16.2) |
| Unknown | 20294 (11.2) | 3810 (9.3) |
| Bleeding |  |  |
| No | 154888 (85.5) | 39486 (96.5) |
| Yes | 2489 (1.4) | 956 (2.3) |
| Unknown | 23756 (13.1) | 477 (1.2) |
| Chest pain |  |  |
| No | 134852 (74.4) | 39155 (95.7) |
| Yes | 25245 (13.9) | 1361 (3.3) |
| Unknown | 21036 (11.6) | 403 (1) |
| Conjunctivitis |  |  |
| No | 148873 (82.2) | 40121 (98) |
| Yes | 563 (0.3) | 51 (0.1) |
| Unknown | 31697 (17.5) | 747 (1.8) |
| Cough |  |  |
| No | 48634 (26.8) | 40919 (100) |
| Yes | 126178 (69.7) |  |
| Unknown | 6321 (3.5) |  |
| Diarrhoea |  |  |
| No | 133616 (73.8) | 38170 (93.3) |
| Yes | 29026 (16) | 2402 (5.9) |
| Unknown | 18491 (10.2) | 347 (0.8) |
| Fatigue malaise |  |  |
| No | 87544 (48.3) | 35861 (87.6) |
| Yes | 69094 (38.1) | 4321 (10.6) |
| Unknown | 24495 (13.5) | 737 (1.8) |
| Headache |  |  |
| No | 131255 (72.5) | 38809 (94.8) |
| Yes | 18260 (10.1) | 1331 (3.3) |
| Unknown | 31618 (17.5) | 779 (1.9) |
| History of fever |  |  |
| No | 52348 (28.9) | 40810 (99.7) |
| Yes | 121688 (67.2) | 106 (0.3) |
| Unknown | 7097 (3.9) | 3 (0) |
| lost altered sense of smell |  |  |
| No | 114205 (63.1) | 37793 (92.4) |
| Yes | 10653 (5.9) | 125 (0.3) |
| Unknown | 56275 (31.1) | 3001 (7.3) |
| Lost altered sense of taste |  |  |
| No | 108974 (60.2) | 37769 (92.3) |
| Yes | 12644 (7) | 80 (0.2) |
| Unknown | 59515 (32.9) | 3070 (7.5) |
| Muscle aches joint pain |  |  |
| No | 119474 (66) | 38594 (94.3) |
| Yes | 30009 (16.6) | 1543 (3.8) |
| Unknown | 31650 (17.5) | 782 (1.9) |
| Runny_nose |  |  |
| No | 139055 (76.8) | 39791 (97.2) |
| Yes | 4592 (2.5) | 286 (0.7) |
| Unknown | 37486 (20.7) | 842 (2.1) |
| Seizures |  |  |
| No | 154421 (85.3) | 36384 (88.9) |
| Yes | 1431 (0.8) | 588 (1.4) |
| Unknown | 25281 (14) | 3947 (9.6) |
| Severe dehydration |  |  |
| No | 62738 (34.6) | 14289 (34.9) |
| Yes | 8923 (4.9) | 1872 (4.6) |
| Unknown | 109472 (60.4) | 24758 (60.5) |
| Shortness of breath |  |  |
| No | 43124 (23.8) | 40919 (100) |
| Yes | 132679 (73.2) |  |
| Unknown | 5330 (2.9) |  |
| Skin rash |  |  |
| No | 149902 (82.8) | 39588 (96.7) |
| Yes | 3551 (2) | 716 (1.7) |
| Unknown | 27680 (15.3) | 615 (1.5) |
| Sore throat |  |  |
| No | 132771 (73.3) | 39583 (96.7) |
| Yes | 11662 (6.4) | 560 (1.4) |
| Unknown | 36700 (20.3) | 776 (1.9) |
| Nausea |  |  |
| No | 132399 (73.1) | 36501 (89.2) |
| Yes | 29681 (16.4) | 4107 (10) |
| Unknown | 19053 (10.5) | 311 (0.8) |
| Wheezing |  |  |
| No | 141083 (77.9) | 40198 (98.2) |
| Yes | 11271 (6.2) | 203 (0.5) |
| Unknown | 28779 (15.9) | 518 (1.3) |

Comorbidities by patients that meet and did not meet the CDC case definition

|  | Case definition met | |
| --- | --- | --- |
| Parameter | Yes (%) | No (%) |
| AIDS/HIV |  |  |
| No | 144654 (89.5) | 33298 (95.8) |
| Yes | 960 (0.6) | 108 (0.3) |
| Unknown | 16056 (9.9) | 1361 (3.9) |
| Asthma |  |  |
| No | 130254 (80.6) | 30988 (89.1) |
| Yes | 20868 (12.9) | 2775 (8) |
| Unknown | 10548 (6.5) | 1004 (2.9) |
| Chronic cardiac disease |  |  |
| No | 113983 (70.5) | 25165 (72.4) |
| Yes | 39171 (24.2) | 8964 (25.8) |
| Unknown | 8516 (5.3) | 638 (1.8) |
| Chronic kidney disease |  |  |
| No | 131047 (81.1) | 29018 (83.5) |
| Yes | 20305 (12.6) | 4770 (13.7) |
| Unknown | 10318 (6.4) | 979 (2.8) |
| Chronic neurological disorder |  |  |
| No | 137026 (84.8) | 29785 (85.7) |
| Yes | 14097 (8.7) | 3958 (11.4) |
| Unknown | 10547 (6.5) | 1024 (2.9) |
| Chronic pulmonary disease |  |  |
| No | 129465 (80.1) | 30853 (88.7) |
| Yes | 23468 (14.5) | 3228 (9.3) |
| Unknown | 8737 (5.4) | 686 (2) |
| Diabetes |  |  |
| No | 110315 (68.2) | 26071 (75) |
| Yes | 40875 (25.3) | 7715 (22.2) |
| Unknown | 10480 (6.5) | 981 (2.8) |
| Hypertension |  |  |
| No | 73644 (45.6) | 20099 (57.8) |
| Yes | 60019 (37.1) | 13683 (39.4) |
| Unknown | 28007 (17.3) | 985 (2.8) |
| Liver disease |  |  |
| No | 149858 (92.7) | 33059 (95.1) |
| Yes | 4622 (2.9) | 1177 (3.4) |
| Unknown | 7190 (4.4) | 531 (1.5) |
| Malignant neoplasm |  |  |
| No | 139358 (86.2) | 30658 (88.2) |
| Yes | 13272 (8.2) | 3404 (9.8) |
| Unknown | 9040 (5.6) | 705 (2) |
| Malnutrition |  |  |
| No | 141645 (87.6) | 31572 (90.8) |
| Yes | 2519 (1.6) | 761 (2.2) |
| Unknown | 17506 (10.8) | 2434 (7) |
| Obesity |  |  |
| No | 115356 (71.4) | 29084 (83.7) |
| Yes | 22432 (13.9) | 2438 (7) |
| Unknown | 23882 (14.8) | 3245 (9.3) |
| Smoking |  |  |
| No | 52008 (32.2) | 9936 (28.6) |
| Yes | 38149 (23.6) | 6331 (18.2) |
| Unknown | 71513 (44.2) | 18500 (53.2) |

Symptoms by patients that meet and did not meet the CDC case definition

|  | Case definition met | |
| --- | --- | --- |
| Parameter | Yes (%) | No (%) |
| Abdominal pain |  |  |
| No | 138632 (85.7) | 32632 (93.9) |
| Yes | 12536 (7.8) | 2125 (6.1) |
| Unknown | 10502 (6.5) | 10 (0) |
| Confusion |  |  |
| No | 123210 (76.2) | 29037 (83.5) |
| Yes | 28162 (17.4) | 2045 (5.9) |
| Unknown | 10298 (6.4) | 3685 (10.6) |
| Bleeding |  |  |
| No | 148170 (91.6) | 34082 (98) |
| Yes | 2295 (1.4) | 664 (1.9) |
| Unknown | 11205 (6.9) | 21 (0.1) |
| Chest pain |  |  |
| No | 128282 (79.3) | 34459 (99.1) |
| Yes | 23943 (14.8) | 297 (0.9) |
| Unknown | 9445 (5.8) | 11 (0) |
| Conjunctivitis |  |  |
| No | 145038 (89.7) | 34711 (99.8) |
| Yes | 492 (0.3) | 40 (0.1) |
| Unknown | 16140 (10) | 16 (0) |
| Cough |  |  |
| No | 45843 (28.4) | 34767 (100) |
| Yes | 113882 (70.4) |  |
| Unknown | 1945 (1.2) |  |
| Diarrhoea |  |  |
| No | 125660 (77.7) | 34000 (97.8) |
| Yes | 28994 (17.9) | 766 (2.2) |
| Unknown | 7016 (4.3) | 1 (0) |
| Fatigue malaise |  |  |
| No | 85968 (53.2) | 33197 (95.5) |
| Yes | 67046 (41.5) | 1564 (4.5) |
| Unknown | 8656 (5.4) | 6 (0) |
| Headache |  |  |
| No | 128142 (79.3) | 34375 (98.9) |
| Yes | 18699 (11.6) | 392 (1.1) |
| Unknown | 14829 (9.2) |  |
| History of fever |  |  |
| No | 53789 (33.3) | 30276 (87.1) |
| Yes | 105605 (65.3) | 4491 (12.9) |
| Unknown | 2276 (1.4) |  |
| lost altered sense of smell |  |  |
| No | 114161 (70.6) | 34734 (99.9) |
| Yes | 10218 (6.3) | 32 (0.1) |
| Unknown | 37291 (23.1) | 1 (0) |
| Lost altered sense of taste |  |  |
| No | 110146 (68.1) | 34754 (100) |
| Yes | 12000 (7.4) | 13 (0) |
| Unknown | 39524 (24.4) |  |
| Muscle aches joint pain |  |  |
| No | 117462 (72.7) | 34309 (98.7) |
| Yes | 30310 (18.7) | 458 (1.3) |
| Unknown | 13898 (8.6) |  |
| Runny_nose |  |  |
| No | 137862 (85.3) | 34558 (99.4) |
| Yes | 4402 (2.7) | 208 (0.6) |
| Unknown | 19406 (12) | 1 (0) |
| Seizures |  |  |
| No | 147544 (91.3) | 30707 (88.3) |
| Yes | 1299 (0.8) | 380 (1.1) |
| Unknown | 12827 (7.9) | 3680 (10.6) |
| Severe dehydration |  |  |
| No | 57860 (35.8) | 11632 (33.5) |
| Yes | 8257 (5.1) | 1093 (3.1) |
| Unknown | 95553 (59.1) | 22042 (63.4) |
| Shortness of breath |  |  |
| No | 40967 (25.3) | 34767 (100) |
| Yes | 119301 (73.8) |  |
| Unknown | 1402 (0.9) |  |
| Skin rash |  |  |
| No | 145222 (89.8) | 34264 (98.6) |
| Yes | 2855 (1.8) | 488 (1.4) |
| Unknown | 13593 (8.4) | 15 (0) |
| Sore throat |  |  |
| No | 131580 (81.4) | 34491 (99.2) |
| Yes | 11615 (7.2) | 276 (0.8) |
| Unknown | 18475 (11.4) |  |
| Nausea |  |  |
| No | 123976 (76.7) | 33220 (95.6) |
| Yes | 30179 (18.7) | 1546 (4.4) |
| Unknown | 7515 (4.6) | 1 (0) |
| Wheezing |  |  |
| No | 137543 (85.1) | 34631 (99.6) |
| Yes | 9681 (6) | 126 (0.4) |
| Unknown | 14446 (8.9) | 10 (0) |

Comorbidities by patients that meet and did not meet the WHO case definition

|  | Case definition met | |
| --- | --- | --- |
| Parameter | Yes (%) | No (%) |
| AIDS/HIV |  |  |
| No | 104332 (87.6) | 84932 (93.9) |
| Yes | 809 (0.7) | 308 (0.3) |
| Unknown | 14018 (11.8) | 5216 (5.8) |
| Asthma |  |  |
| No | 93695 (78.6) | 77908 (86.1) |
| Yes | 15973 (13.4) | 8934 (9.9) |
| Unknown | 9491 (8) | 3614 (4) |
| Chronic cardiac disease |  |  |
| No | 85776 (72) | 61302 (67.8) |
| Yes | 26011 (21.8) | 26698 (29.5) |
| Unknown | 7372 (6.2) | 2456 (2.7) |
| Chronic kidney disease |  |  |
| No | 96343 (80.9) | 73031 (80.7) |
| Yes | 13583 (11.4) | 14012 (15.5) |
| Unknown | 9233 (7.7) | 3413 (3.8) |
| Chronic neurological disorder |  |  |
| No | 100080 (84) | 76448 (84.5) |
| Yes | 9665 (8.1) | 10428 (11.5) |
| Unknown | 9414 (7.9) | 3580 (4) |
| Chronic pulmonary disease |  |  |
| No | 95187 (79.9) | 75284 (83.2) |
| Yes | 16331 (13.7) | 12581 (13.9) |
| Unknown | 7641 (6.4) | 2591 (2.9) |
| Diabetes |  |  |
| No | 80176 (67.3) | 64495 (71.3) |
| Yes | 29929 (25.1) | 22611 (25) |
| Unknown | 9054 (7.6) | 3350 (3.7) |
| Hypertension |  |  |
| No | 53392 (44.8) | 43409 (48) |
| Yes | 42399 (35.6) | 34903 (38.6) |
| Unknown | 23368 (19.6) | 12144 (13.4) |
| Liver disease |  |  |
| No | 109938 (92.3) | 85402 (94.4) |
| Yes | 3204 (2.7) | 3081 (3.4) |
| Unknown | 6017 (5) | 1973 (2.2) |
| Malignant neoplasm |  |  |
| No | 102016 (85.6) | 78581 (86.9) |
| Yes | 9168 (7.7) | 9140 (10.1) |
| Unknown | 7975 (6.7) | 2735 (3) |
| Malnutrition |  |  |
| No | 102571 (86.1) | 80428 (88.9) |
| Yes | 1617 (1.4) | 2103 (2.3) |
| Unknown | 14971 (12.6) | 7925 (8.8) |
| Obesity |  |  |
| No | 81072 (68) | 72015 (79.6) |
| Yes | 18238 (15.3) | 7828 (8.7) |
| Unknown | 19849 (16.7) | 10613 (11.7) |
| Smoking |  |  |
| No | 40901 (34.3) | 25445 (28.1) |
| Yes | 28274 (23.7) | 19557 (21.6) |
| Unknown | 49984 (41.9) | 45454 (50.2) |

Symptoms by patients that meet and did not meet the WHO case definition

|  | Case definition met | |
| --- | --- | --- |
| Parameter | Yes (%) | No (%) |
| Abdominal pain |  |  |
| No | 96712 (81.2) | 81396 (90) |
| Yes | 9368 (7.9) | 6360 (7) |
| Unknown | 13079 (11) | 2700 (3) |
| Confusion |  |  |
| No | 87937 (73.8) | 69965 (77.3) |
| Yes | 20279 (17) | 13858 (15.3) |
| Unknown | 10943 (9.2) | 6633 (7.3) |
| Bleeding |  |  |
| No | 104304 (87.5) | 85467 (94.5) |
| Yes | 1295 (1.1) | 2031 (2.2) |
| Unknown | 13560 (11.4) | 2958 (3.3) |
| Chest pain |  |  |
| No | 88849 (74.6) | 81384 (90) |
| Yes | 18548 (15.6) | 6440 (7.1) |
| Unknown | 11762 (9.9) | 2632 (2.9) |
| Conjunctivitis |  |  |
| No | 99587 (83.6) | 86315 (95.4) |
| Yes | 425 (0.4) | 154 (0.2) |
| Unknown | 19147 (16.1) | 3987 (4.4) |
| Cough |  |  |
| No | 19166 (16.1) | 68800 (76.1) |
| Yes | 99022 (83.1) | 21292 (23.5) |
| Unknown | 971 (0.8) | 364 (0.4) |
| Diarrhoea |  |  |
| No | 84807 (71.2) | 82806 (91.5) |
| Yes | 24695 (20.7) | 5579 (6.2) |
| Unknown | 9657 (8.1) | 2071 (2.3) |
| Fatigue malaise |  |  |
| No | 48913 (41) | 73828 (81.6) |
| Yes | 56939 (47.8) | 13128 (14.5) |
| Unknown | 13307 (11.2) | 3500 (3.9) |
| Headache |  |  |
| No | 84196 (70.7) | 83740 (92.6) |
| Yes | 16195 (13.6) | 2760 (3.1) |
| Unknown | 18768 (15.8) | 3956 (4.4) |
| History of fever |  |  |
| No | 15781 (13.2) | 76495 (84.6) |
| Yes | 103229 (86.6) | 13848 (15.3) |
| Unknown | 149 (0.1) | 113 (0.1) |
| lost altered sense of smell |  |  |
| No | 74613 (62.6) | 74683 (82.6) |
| Yes | 8835 (7.4) | 1437 (1.6) |
| Unknown | 35711 (30) | 14336 (15.8) |
| Lost altered sense of taste |  |  |
| No | 70596 (59.2) | 74054 (81.9) |
| Yes | 10431 (8.8) | 1613 (1.8) |
| Unknown | 38132 (32) | 14789 (16.3) |
| Muscle aches joint pain |  |  |
| No | 74165 (62.2) | 82308 (91) |
| Yes | 26509 (22.2) | 4193 (4.6) |
| Unknown | 18485 (15.5) | 3955 (4.4) |
| Runny_nose |  |  |
| No | 92006 (77.2) | 85279 (94.3) |
| Yes | 3884 (3.3) | 844 (0.9) |
| Unknown | 23269 (19.5) | 4333 (4.8) |
| Seizures |  |  |
| No | 104350 (87.6) | 81801 (90.4) |
| Yes | 811 (0.7) | 1091 (1.2) |
| Unknown | 13998 (11.7) | 7564 (8.4) |
| Severe dehydration |  |  |
| No | 42314 (35.5) | 31877 (35.2) |
| Yes | 6307 (5.3) | 3865 (4.3) |
| Unknown | 70538 (59.2) | 54714 (60.5) |
| Shortness of breath |  |  |
| No | 23583 (19.8) | 59174 (65.4) |
| Yes | 93309 (78.3) | 30488 (33.7) |
| Unknown | 2267 (1.9) | 794 (0.9) |
| Skin rash |  |  |
| No | 100712 (84.5) | 85229 (94.2) |
| Yes | 2186 (1.8) | 1742 (1.9) |
| Unknown | 16261 (13.6) | 3485 (3.9) |
| Sore throat |  |  |
| No | 86519 (72.6) | 84419 (93.3) |
| Yes | 10047 (8.4) | 1841 (2) |
| Unknown | 22593 (19) | 4196 (4.6) |
| Nausea |  |  |
| No | 84046 (70.5) | 80668 (89.2) |
| Yes | 24849 (20.9) | 7642 (8.4) |
| Unknown | 10264 (8.6) | 2146 (2.4) |
| Wheezing |  |  |
| No | 94132 (79) | 84451 (93.4) |
| Yes | 7951 (6.7) | 2671 (3) |
| Unknown | 17076 (14.3) | 3334 (3.7) |
